# Long‐term persistence of horse fecal DNA in the environment makes equids particularly good candidates for noninvasive sampling

**DOI:** 10.1002/ece3.3956

**Published:** 2018-03-26

**Authors:** Sarah R. B. King, Kathryn A. Schoenecker, Jennifer A. Fike, Sara J. Oyler‐McCance

**Affiliations:** ^1^ Natural Resource Ecology Laboratory Department of Ecosystem Science and Sustainability Colorado State University Fort Collins CO USA; ^2^ United States Geological Survey Fort Collins Science Center Fort Collins CO USA

**Keywords:** conservation, *Equus*, genotype, horse, noninvasive sampling

## Abstract

Fecal DNA collected noninvasively can provide valuable information about genetic and ecological characteristics. This approach has rarely been used for equids, despite the need for conservation of endangered species and management of abundant feral populations. We examined factors affecting the efficacy of using equid fecal samples for conservation genetics. First, we evaluated two fecal collection methods (paper bag vs. ethanol). Then, we investigated how time since deposition and month of collection impacted microsatellite amplification success and genotyping errors. Between May and November 2014, we collected feral horse fecal samples of known age each month in a feral horse Herd Management Area in western Colorado and documented deterioration in the field with photographs. Samples collected and dried in paper bags had significantly higher amplification rates than those collected and stored in ethanol. There was little difference in the number of loci that amplified per sample between fresh fecal piles and those that had been exposed to the environment for up to 2 months (in samples collected in paper bags). After 2 months of exposure, amplification success declined. When comparing fresh (0–2 months) and old (3–6 months) fecal piles, samples from fresh piles had more matching genotypes across samples, better amplification success and less allelic dropout. Samples defecated during the summer and collected within 2 months of deposition had highest number of genotypes matching among samples, and lowest rates of amplification failure and allelic dropout. Due to the digestive system and amount of fecal material produced by equids, as well as their occurrence in arid ecosystems, we suggest that they are particularly good candidates for noninvasive sampling using fecal DNA.

## 1 INTRODUCTION

Noninvasive methods such as the use of DNA extracted from fecal samples are increasingly being used to examine occupancy, population size, diet, and even hormones of a wide range of species (Bowser, Diamond, & Addison, 2013; Ernest, Penedo, May, Syvanen, & Boyce, 2000; Macandza, Owen‐Smith, & Le Roux, 2014; Oyler‐ McCance et al., 2018; Powell & Monfort, 2001; Prugh, Ritland, Arthur, & Krebs, 2005; Schoenecker et al., 2014). Noninvasive sampling of fecal DNA has been used for conservation goals as varied as estimating population sizes of forest elephants (*Loxodonta cyclosis*, Eggert, Eggert, & Woodruff, 2003; Eggert et al., 2013), identifying hybrids in a reintroduced population of red wolves (*Canis rufus*, Adams, Kelly, & Waits, 2003), intra‐community relationships of bonobos (*Pan paniscus,* Gerloff, Hartung, Fruth, Hohmann, & Tautz, 1999), and use of resources by individual Sonoran pronghorn (*Antilocapra americana sonoriensis*, Woodruff, Lukacs, Christianson, & Waits, 2016). Several studies have highlighted the advantages of noninvasive sampling compared to traditional methods (Beja‐Pereira, Oliveira, Alves, Schwartz, & Luikart, 2009; Kohn & Wayne, 1997; Luikart, Ryman, Tallmon, Schwartz, & Allendorf, 2010), and in some cases they have been shown to be at least as effective at monitoring various parameters, if not more so, than traditional methods. The promise of these noninvasive approaches has yet to be fully realized and extends to a wide variety of species for which demographic and other ecological information is needed.

Five of the seven extant equid species are threatened with extinction (Moehlman, King, & Kebede, 2016). Conversely, the two domesticated equids (horses, *Equus ferus caballus*, and donkeys, *E. africanus asinus*) are abundant in feral populations to the point of being considered nuisance species in some places (Garrott & Oli, 2013; Woolnough et al., 2012). For both rare and common equids, conservation and management can be greatly enhanced by understanding genetic characteristics of populations and individuals. Unlike ruminants, equids are bulk feeders which consume high quantities of relatively low‐quality forage to meet their nutritional needs (Schoenecker, King, Nordquist, Nandintsetseg, & Cao, 2016). This results in production of large quantities of fecal matter rich in epithelial cells from passage through the gut, making them potentially very good candidates for fecal analyses. Fecal DNA has been used to inform the conservation of some endangered equid species: the African wild ass (*E. africanus*, Rosenbom, Costa, Steck, Moehlman, & Beja‐Pereira, 2011), Przewalski's horse (*E. ferus przewalskii*, Liu et al., 2014a), and Grevy's zebra, (*E. grevyi*, Kebede et al., 2014). However, noninvasive sampling has been more commonly used for carnivores than herbivores (Harris et al., 2010; Poole, Reynolds, Mowat, & Paetkau, 2011), and techniques have mostly been tested on carnivores (e.g., Murphy, Kendall, Robinson, & Waits, 2007). Thus, sample collection techniques for herbivores need to be tested and optimized, as factors affecting amplification success of fecal DNA may be different in this group.

For conservation and management of wildlife and feral animals there, is a need for consistent, inexpensive, and simple methods of noninvasive sampling for genetic analysis. Ideally, such sampling methods could be explained to personnel with no scientific training, using supplies that are readily available. Equids produce large amounts of fecal material that can be found easily, making this source of DNA easier to collect than hair. Fecal samples are typically placed in ethanol, with some studies reporting that this method of preservation produced better amplification results than when collected in DMSO/EDTA/Tris/salt (DETs) buffer (e.g., Panasci et al., 2011). Additional sample collection methods (other than ethanol and DETs buffer) include freezing samples in plastic bags or drying them with silica either immediately or after initial collection in alcohol (Frantzen, Silk, Ferguson, Wayne, & Kohn, 1998; Hettinga et al., 2012; Murphy et al., 2002; Nsubuga et al., 2004; Panasci et al., 2011; Wasser, Houston, Koehler, Cadd, & Fain, 1997). The results of studies have been mixed in terms of which sampling methods produce the best results (Renan et al., 2012), and there may be a species‐ or genus‐specific relationship between collection method and DNA amplification success.

In addition to variation in amplification effectiveness by species or collection method, successful amplification and genotyping of fecal DNA depends on time since deposition and environmental factors. Amplification success was reported to be higher in the colder temperatures of winter for several studies (Harris et al., 2010; Hettinga et al., 2012; Liu et al., 2014b; Lucchini et al., 2002; Maudet, Luikart, Dubray, Von Hardenberg, & Taberlet, 2004). In general, fecal pellets persist longer in dry environments or where they are sheltered from rain (Harestad & Bunnell, 1987). While aridity may preserve DNA, UV light will degrade it (Ravanat, Douki, & Cadet, 2001), thus fecal samples that have had prolonged exposure to sunlight may be less likely to yield amplifiable DNA. In addition, amplification success is affected by time since deposition and ambient temperature, as well as dew point (Murphy et al., 2007). Collecting fresh fecal samples reduces the impacts of these environmental effects, with several studies demonstrating less successful amplification of older feces (Foran, Crooks, & Minta, 1997; Lucchini et al., 2002; Maudet et al., 2004; Piggott, 2004; Poole et al., 2011). Desiccation rates of feces can vary during different times of the year, making assessment of age in the field difficult (Stenglein, Waits, Ausband, Zager, & Mack, 2010). Collecting very fresh fecal samples can be time‐consuming, as it requires knowledge of where the animals are likely to have been or even direct observation of the individual. Sampling only areas of known use by a target species may bias population estimates. Therefore, if older feces can yield amplifiable DNA, then a greater number of samples can potentially be collected from a wider or more randomly chosen area.

We conducted research to assess whether it would be feasible to use fecal DNA to examine various parameters of feral horse ecology and population biology. The objectives of this study were to compare two techniques for collecting feral horse fecal samples (paper bags and ethanol vials); investigate the relationship between fecal pile age and genotyping success, and the optimal time of year to collect samples (through both amplification success and genotyping errors); and develop a visual guide documenting horse feces deterioration over time in the environment to aid in field collection. We expected to see some difference in amplification success and genotyping errors between the collection methods, that as samples aged there would be a rapid decline in amplification success, and that the optimal time to collect samples would be in cool or dry times of the year.

## 2 MATERIALS AND METHODS

### 2.1 Study area

**Figure 1:**
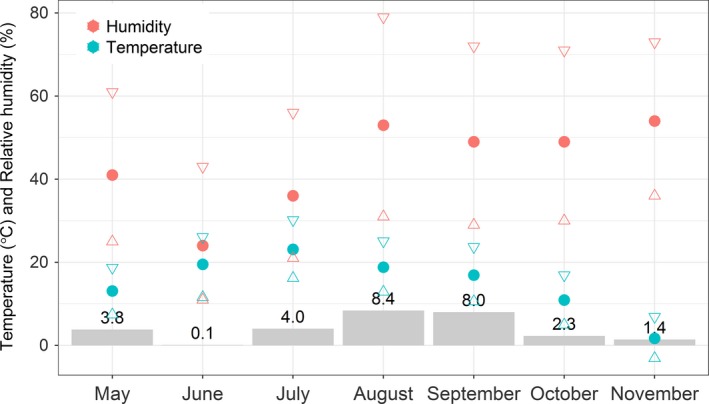
Weather data from the Pine Ridge National Oceanic and Atmospheric Administration (NOAA) Remote Automatic Weather Station (RAWS), located 1 km east of Little Book Cliffs Herd Management Area, Colorado, USA. Gray bars show the total precipitation (cm) each month during the 2014 study period. Mean temperature (°C) and relative humidity (%) for each month are shown by solid circles; mean maximum and minimum values are shown by triangles

We conducted our study at the 146 km^2^ Little Book Cliffs Wild Horse Herd Management Area, near Grand Junction, Colorado, USA, between May and November 2014. The area mostly consists of mesa top covered in pinyon pine (*Pinus edulis*) and juniper (*Juniperus* spp.) interspersed with sagebrush (*Artemisia* spp.) and grass meadows. The area is dissected by deep canyons, some of which have grass and water along the bottom. Water sources for horses and wildlife are located throughout the area as natural creeks or maintained in tanks. Data from the Pine Ridge National Oceanic and Atmospheric Administration (NOAA) Remote Automatic Weather Station (RAWS), located on a mesa adjacent to the study site, showed that during our study period conditions were mostly dry, with the exception of 8 cm of precipitation falling in both August and September (Figure 1).

### 2.2 Sample collection

We took samples from fecal piles deposited in meadows in the north of the study area (an area known as North Soda) because of accessibility and to retain consistency. In May 2014, we found 20 fecal piles that were either observed being defecated or were determined to be very fresh (still moist on the outside and attracting flies). We marked each pile with a numbered stake, took a photograph, recorded GPS coordinates, and took samples from a random 10 of the 20 piles. In June, we re‐visited and photographed the 20 piles marked in May and sampled a random 10 of the May piles. We marked twenty fresh piles from June as in May, and sampled a random 10 of the June piles. This was repeated every month until November: each month 20 fresh piles were marked with a stake, photographed, and GPS location recorded with a random 10 sampled; plus all of the 20 piles marked in every previous month were visited and photographed, with samples taken randomly from 10. New fecal piles close to a marked pile could be detected by their relative freshness; if they were directly adjacent to a marked pile we sampled from the opposite side. Our sampling strategy (sampling a random 10 piles of the 20 marked piles) ensured that a sufficient amount of the pile would remain at the end of the study; thus, not all piles were sampled every month. In most months, it was possible to find 20 fresh piles within the same day. However in May, June, and October it was not possible to find 20 fresh piles on the same day, so some piles from these months were separated in age by one to 5 days (mean 30 ± 3 days SD between sample collection points overall). Each pile was fresh on the day it was first sampled, photographed, and marked. Deterioration of fecal piles over time was documented with digital photographs.

Fecal samples were collected and stored in two ways: paper bags, and vials of 95% ethanol. We avoided contamination of samples using nitrile gloves or sterile tongue depressors to manipulate the fecal bolus. For paper bag samples, we placed a whole fecal bolus in a paper lunch bag with details of the sample written on it (sample number, collector, area, date). We then placed these paper bag samples in large cotton bags to hang and dry during the sampling session. Three to 5 days after collection, we transferred samples to a drying oven to desiccate at 40°C for 3 days. We collected samples in ethanol from July onwards: we placed approximately 2 cm^3^ of a bolus from the same pile as the paper bag sample in to a vial of ethanol (estimating a ratio of 1:3 to 1:4 feces to alcohol within each vial). Vials of ethanol were kept at room temperature and stored upright to prevent leakage.

### 2.3 Molecular methods

We cut a small amount of feces (~5 mm^3^, or enough to fill about 1 ml of a 1.7 ml tube) from the outer layer of a sample for analyses. We retained the rest of the sample for other tests. Genomic DNA was isolated from horse feces following the animal tissue protocol of the DNeasy96 Blood and Tissue kit (Qiagen Inc., Valencia, CA) with the following modifications: (1) samples were incubated overnight at 56°C in 900 μl Buffer ATL with 20 μl proteinase K and 20 μl 1M DTT; and (2) DNA was eluted in 80 μl Buffer AE. Extraction negatives were included with every set of extractions. Samples were amplified across eight variable microsatellite loci (AHT4: called HMB4 in Binns, Holmes, Holliman, and Scott (1995), HMS1: Guérin, Bertaud, and Amigues (1994), HTG4 and HTG6: Ellegren, Johansson, Sandberg, and Andersson (1992), and HMS3, HMS6, HMS7, ASB2: redesigned primers from Eggert et al. (2010)), which were chosen because they were highly polymorphic, worked well with fecal DNA, and were easy to score. These markers were tested to ensure that they had a low probability of identity (i.e., the probability that two individuals would have the same genotype (Waits, Luikart, & Taberlet, 2001): using the GenAlex Excel add‐in (Peakall & Smouse, 2006, 2012) we determined that this was true at five microsatellites that were least polymorphic (*p* < .001). The eight microsatellites were amplified using the preamplification method described by Piggott, Bellemain, Taberlet, and Taylor (2004). The preamplification method is a two‐step procedure that involved an initial PCR using a pool of unlabeled primer pairs for all eight loci. This initial step was performed following the conditions outlined in Piggott et al. (2004) with the exception of using 10 μl fecal DNA as the template for the 50‐μl reaction. The second step used 3 μl of the PCR product produced in the first step as template for 12.5 μl reactions containing 0.2 mmol/L of each dNTP, 1× GoTaq Flexi Buffer (Promega), 1.5 mmol/L MgCl_2_, 1× BSA, 0.5 ⋅mol/L of each primer (dye‐labeled forward), and 1 U of *Taq* DNA polymerase (Promega). The amplification conditions for the second step were as follows: 94°C for 2 min, then 94°C for 1 min, annealing temperature (55°C: ASB2, HTG4, HTG6; 59°C: HMS6; 60°C: AHT4, HMS3, HMS7, HMS1) for 1 min, 72°C for 1 min for 40 cycles, followed by 72°C for 10 min, and a final extension at 60°C for 45 min. We ran positive and negative controls during all PCR amplifications. PCR products were multi‐loaded based on product size and primer label, combined with GeneScan LIZ 600 internal lane size standard (Applied Biosystems), and electrophoresed through a capillary gel matrix using an AB3500 Automated DNA Sequencer (Applied Biosystems). Allele sizes were determined for each locus using GeneMapper v5 software (Applied Biosystems).

Fecal samples can have lower quantities of DNA that is often of lower quality, both factors that increase the probability of genotyping error (Taberlet, Waits, & Luikart, 1999). To account for such errors, DNA extracted from a fecal sample can be amplified multiple times and genotypes from those amplifications compared to identify and quantify errors within that sample. In addition, multiple samples from the same fecal pile sampled at different times as deposition can be amplified and genotypes compared to determine whether errors are more prevalent in older samples. Potential errors can include false alleles (where the genotype obtained is incorrect due to PCR errors, problems with electrophoresis, or human error) or allelic dropout (where one allele of a heterozygote does not amplify) and comparisons among amplifications within a sample can help identify these errors and determine the correct genotype (Broquet & Petit, 2004). To minimize error for each DNA extraction, each sample was PCR‐amplified multiple times (at least twice but often three, four, or five times) at every microsatellite locus and we compared genotypes across these multiple amplifications. If a sample had matching genotypes in at least two amplifications, that matching genotype was retained. Samples that did not have matching genotypes after multiple amplifications or that amplified only once were treated as missing data at that locus. Genotypes were scored by two independent observers. If a sample failed to amplify or was scored differently between the two attempts, it was re‐amplified until two matching genotypes were obtained or the DNA was depleted.

### 2.4 Data analyses

#### 2.4.1 Comparison of collection method

To compare paper bag and ethanol samples, we only used fecal samples from the same fecal pile that that were collected and stored using both methods. We examined amplification success in several ways. First, within each collection method, we counted the number of samples that had successful amplification at all eight loci. Further, since we knew that we needed amplification at a minimum of five loci for unique identification, we determined amplification rates for only those samples with genotypes at five or more loci. Finally, we counted the number of samples that did not amplify at any loci and compared those results between collection methods. Paired Student's *t*‐tests were used to determine statistical significance of results.

#### 2.4.2 Comparison of amplification success through time

Because samples collected in paper bags had higher amplification success (see Results section) and because sample collection in ethanol did not span the entire summer, we examined the relationship between amplification success and age of fecal pile using only samples collected in paper bags. For this analysis, we sorted samples by age of fecal pile (months since time of deposition) when the sample was collected. We then calculated number of loci that successfully amplified for each sample in each fecal age group. We tested effect of age on amplification success using ANOVA with fecal pile age as a discrete variable.

#### 2.4.3 Comparing genotyping error between fresh and old samples

To investigate whether genotyping error was higher in older samples, we again used only samples collected in paper bags, and removed any samples that had genotypes at fewer than five loci. Most marked fecal piles had multiple samples taken through time (the piles were of various age). For each fecal pile, we separated the data into two groups: fresh (0–2 months old) and old (3 – 6 months old). We then compared the genotypes for the eight loci among the two or three samples within each group (fresh vs. old) for each fecal pile. We counted instances of allelic dropout (where one sample was a heterozygote and one or more samples were homozygous for one of the two matching alleles), missing data (failure to amplify), false alleles (three or more alleles present), and matching genotypes. Differences were examined using Student's *t*‐tests.

#### 2.4.4 Comparison of error rates of fresh samples deposited in different months of the year

As temperature and precipitation vary considerably from May to November in our study area, and because the diet of horses shifts throughout the summer, we tested whether genotyping error rates differed between fecal piles deposited in different months. To examine this, we used only those samples collected in paper bags that were fresh (0–2 months old), removing any samples that had genotypes at fewer than five loci. We compared the genotypes for the eight loci among the two or three samples from each fecal pile and tested differences among months in genotyping error (allelic dropout, missing data, false alleles) and matching genotypes using ANOVA.

## 3 RESULTS

### 3.1 Subjective description of deterioration of fecal piles

There was a noticeable difference in color and consistency between fecal piles deposited in May, June, and July, and those deposited in September, October, and November (Table 1). In the spring/early summer, fecal boli were green and less formed than those deposited later in the year. This appears to have affected how they deteriorated, as the boli that were less formed when deposited turned white and desiccated over 3 months, whereas boli that were more formed when deposited remained solid and a darker color for longer.

**Table 1:** Subjective description of fecal pile appearance and deterioration at Little Book Cliffs Wild Horse Herd Management Area, Colorado, USA between May and November. Photographs demonstrate the deterioration of the same pile over time

Month	Fresh	1 month	2 months	3 months	4 months	5 months	6 months
May	Often loose (some or all boli not formed), bright greenish brown 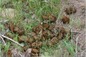	Brownish white, intact, boli often stuck together 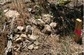	Whitish brown, intact, often boli are stuck together 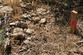	Mostly white, intact 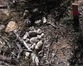	White, intact 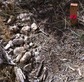	White, beginning to decay 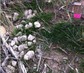	White, decaying, often scattered 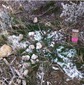
June	Sometimes loose, bright greenish brown to bright brown 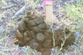	Brown, intact, boli often stuck together 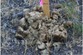	Brownish white, intact, boli often stuck together 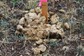	Brownish white to whitish brown, intact 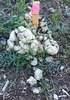	Whitish brown, intact, sometimes scattered 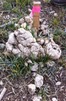	Whitish brown to white, beginning to decay 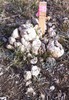	
July	Occasionally loose, bright brownish green 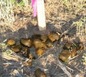	Brown, intact 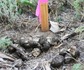	Dull brown with white patches 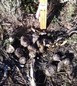	Brown to whitish brown with some brown patches 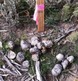	Whitish brown to white, may be scattered 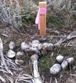		
August	Bright brown 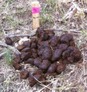	Brown, intact 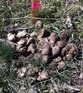	Dull brown with white patches 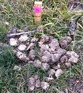	Brown to whitish brown with some brown patches 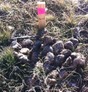			
September	Bright brown 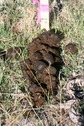	Brown, intact 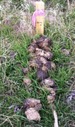	Dull brown with white patches 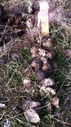				
October	Dark brown 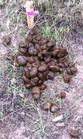	Dried dark brown, intact 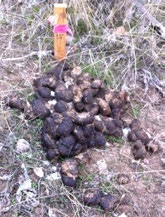					
November	Dark brown 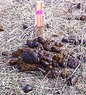						

### 3.2 Comparison of collection method

Between July and November 2014, 250 samples were collected from 120 fecal piles in both paper bags and ethanol vials. Samples collected in paper bags had far greater amplification success than those collected in ethanol vials (Student's *t* = 11.896, df = 249, *p* = <.0001; Table 2). Over half (55.6%) of paper bag samples amplified at all eight loci compared to only 17.2% of ethanol samples. When we consider only samples with genotypes for at least five loci (the number of loci needed to uniquely identify individuals), 90% of the samples collected in paper bags were usable compared to only 55.2% of those that were collected in ethanol. The complete failure to amplify at any locus was higher (17.6%) for ethanol samples compared to only 2.8% of samples collected in paper bags.

**Table 2:** Amplification success of samples collected from horse fecal piles in both paper bags and vials of ethanol at Little Book Cliffs Herd Management Area, Colorado, USA. Number of samples that amplified at ≥5 loci is given as the probability of identity (P_ID_) was ≤0.001 when at least five loci were included for both collection methods

	Paper bags (*N* = 250)	Ethanol vials (*N* = 250)
Amplified at 8 loci (%)	139 (55.6)	43 (17.2)
Amplified at ≥5 loci (%)	225 (90)	138 (55.2)
Did not amplify at any loci (%)	7 (2.8)	44 (17.6)

### 3.3 Effect of fecal pile age and month of collection

**Figure 2:**
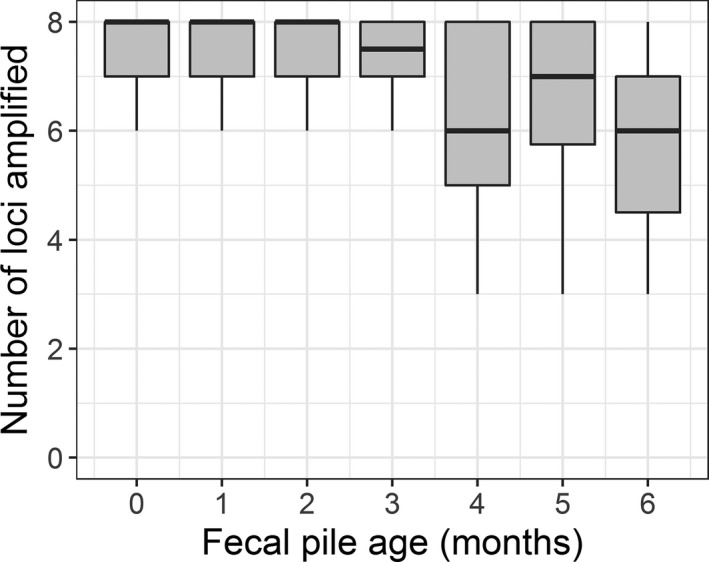
Boxplot showing the number of loci that amplified from samples collected in paper bags from feral horse fecal piles at Little Book Cliffs Herd Management Area, Colorado, USA when they were fresh (0 months) to 6 months old

**Figure 3:**
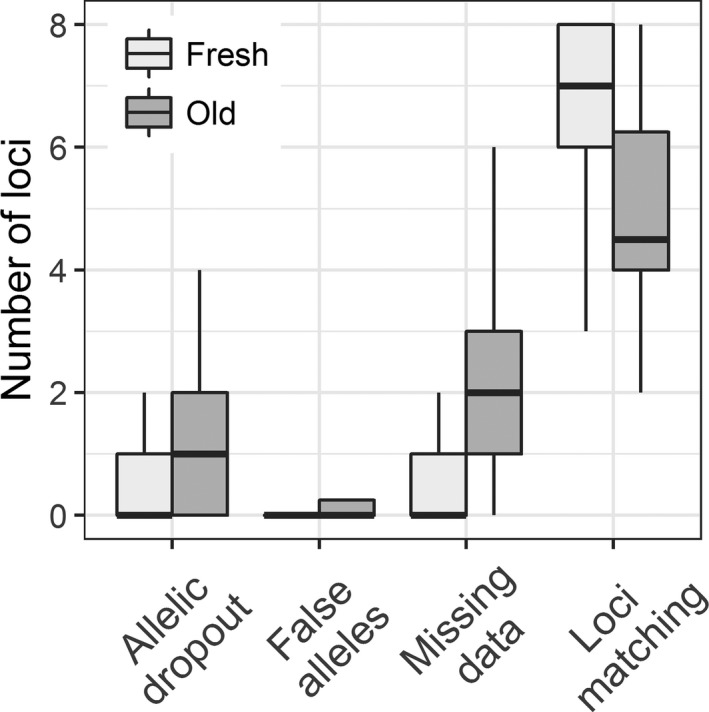
Difference in genotyping errors between samples from fresh fecal piles (0–2 months old) and old fecal piles (3–6 months old) collected in paper bags from feral horse fecal piles at Little Book Cliffs Herd Management Area, Colorado, USA. Loci matching is the number of loci that matched (out of a total of eight loci) in a genotype of multiple samples taken from the same fecal pile

Using the full data set from paper bag samples (May to November, *n* = 280 samples), we found little difference in the number of loci that amplified per sample between samples from fresh piles and those that had been exposed to the environment for up to 2 months. After this, there was a decline in amplification success (ANOVA *F*_1,278_ = 22.49, *p* = <.0001; Figure 2), but even the relatively small number of samples (*n* = 10) from fecal piles that were 6 months old amplified at six of the eight loci on average. As our samples came from unknown individuals we could not definitively assess genotyping success (i.e., we did not know the true genotype), but one would expect multiple samples collected from the same fecal pile over time to have the same genotype. When looking at repeated samples from the same fecal pile, samples from fresher piles (0–2 months old) had more matching genotypes across samples (*t* = 3.546, df = 39.816, *p* = .001), better amplification success (i.e., less missing data at a locus; *t* = −3.6881, df = 31.141, *p* = .0009), and less allelic dropout (*t* = −2.5935, df = 33.029, *p* = .01405) than samples from older piles (3–6 months old; Figure 3). The number of false alleles were similar between samples from fresh and old piles (*t* = −0.41715, df = 55.966, *p* = .6782).

**Figure 4:**
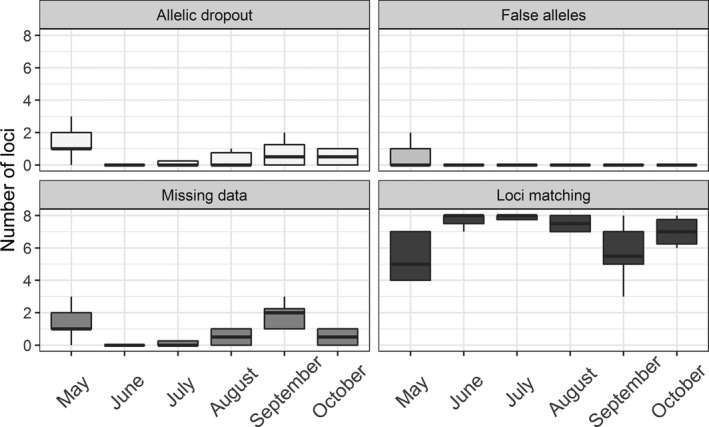
Comparison of error rate in samples deposited in different months of the year (2014) and collected in paper bags from feral horse fecal piles at Little Book Cliffs Herd Management Area, Colorado, USA. November is excluded as there were no repeat samples from piles in that month

We used these repeated samples from fresh piles (0–2 months old) to assess which month was best for equid fecal sample collection. Samples deposited in June, July, and August tended to perform best on the four metrics we tested (Figure 4). May and September had the highest rates of missing data (*F*_5,46_ = 6.492, *p* = .000121), with May also having high rates of allelic dropout (*F*_5,46_ = 3.109, *p* = .01686) and lowest number of genotypes matching among samples (*F*_5,46_ = 2.617, *p* = .03648). Rates of false alleles were low, with no statistical difference among months (*F*_5,46_ = 0.7195, *p* = .6121), but they appear greater in samples from May (Figure 4).

## 4 DISCUSSION

As only a few studies have been conducted on equids using noninvasively collected fecal DNA (Kebede et al., 2014; Liu et al., 2014a; Rosenbom et al., 2011), our study to assess the feasibility of using such methods along with an optimization of collection methods and quantification of genotyping errors could be used as a guide for subsequent efforts. Most published noninvasive studies of mammals collected and stored fecal samples in ethanol (Adams & Waits, 2006; Brinkman, Schwartz, Person, Pilgrim, & Hundertmark, 2009; De Barba et al., 2010; Eggert et al., 2003; Gerloff et al., 1999; Harris et al., 2010; Maudet et al., 2004; Valiere et al., 2006). Some comparison studies found ethanol to be the most effective method for preserving fecal DNA (Murphy et al., 2002; Panasci et al., 2011), although their comparison tests did not include collecting and drying samples in paper bags. Liu et al. (2014c) found ethanol to be the most effective preservative of Przewalski's horse fecal DNA, in comparison with drying and freezing the fecal sample. We were therefore somewhat surprised to find that for feral horses, collecting and storing fecal samples in paper bags was much more successful than ethanol. Paper bags have been used to gather fecal samples for genetic data in a few other studies (Piggott, 2004; Piggott & Taylor, 2003; Poole et al., 2011), including for equids (Kebede et al., 2014; Rosenbom et al., 2011), but to our knowledge this is the first study to compare the amplification of DNA from fecal samples collected in ethanol to those collected in paper bags. Renan et al. (2012) found an interaction between the collection and preservation method and the method used to extract DNA from Asiatic wild ass (*Equus hemionus*) fecal samples, and also found ethanol (and subsequent drying with silica) to be least effective.

Paper bags are less expensive than alcohol and vials: at the time of our study (2014) paper bags cost about $0.04 per sample, whereas the cost of 25 ml of 95% ethanol plus a 50‐ml conical tube was about $1.89 per sample. While paper bag samples needed careful drying, they only required space for storage. In contrast, ethanol should be stored in a flammable cabinet and there are various regulations controlling shipping of it. Further disadvantages of collecting samples in ethanol are the risks of spills erasing sample information from the tube, and the weight of carrying it in the field. The only potential complication of collecting samples in paper bags is that very fresh samples may need to be double‐bagged to avoid seepage and contamination of other samples. Paper bags not only resulted in better genetic data than vials of ethanol, but are simpler and less expensive for collection of equine fecal samples. As such, this method is ideal for field staff both in remote locations in Asia and Africa where wild equids occur, and for managers of feral horse and burro populations who may not have access to laboratory supplies, or who spend extensive periods in remote field settings.

Most authors have advocated for fecal samples to be collected as soon after deposition as possible in order to prevent degradation of DNA due to weather and ultraviolet radiation (e.g., Foran et al., 1997). There was a significant increase in DNA amplification errors five to 7 days after defecation in three species where it was tested (coyote (*C. latrans*), Panasci et al., 2011; snowshoe hares (*Lepus americanus*), Cheng, Hodges, Sollmann, & Mills, 2017; Sitka black‐tailed deer (*Odocoileus hemionus sitkinensis*), Brinkman et al., 2009). Although DNA was amplified from brown bear (*Ursos arctos*) scat after being exposed to the environment for 60 days, Murphy et al. (2007) found the most significant decline in amplification success was after the first 2 days. In marked contrast to these studies, horse feces had good amplification success (amplification at >90% of loci) and low error rates until it had been in the environment for longer than 2 months. This indicates that it is not necessary to collect only the freshest feces for feral horses and likely other equid species. Removing the constraint of collecting fresh feces has ramifications for sampling design, as surveys for collecting equid fecal samples can be carried out using transects or a randomized design to avoid detection bias, rather than focusing around water holes, trails, or other areas where animals are known to congregate.

During our study, there was variation in rainfall and temperature among the months, with the climate being mostly dry. We expected the best genetic results to be from samples deposited in the coldest or driest months, as in other studies (Liu et al., 2014b; Lucchini et al., 2002; Maudet et al., 2004; Nsubuga et al., 2004). Our results indicated that samples from fecal piles deposited from June to August and collected when fresh (≤2 months old) provided the best genetic data. Although August was one of the wettest months of our study it was also one of the warmest. Thus, in the arid western United States, we would recommend collecting fecal samples that are less than 2 months old in late summer, and in other locations during a hot dry season. Fecal piles deposited in May had a high incidence of mold, both in the field and in collected samples. This could potentially explain why samples deposited in May had higher rates of amplification failure and genotyping error. Qualitative evaluation of fecal piles also indicated that sample collection is likely to be most efficient during mid to late summer, as feces are more formed on deposition and remain a brown color for longer. It is therefore possible that a combination of a transition to a drier diet from fresh spring grass, and warmer weather potentially drying the fecal sample quickly despite rain, contributed to better preservation of fecal DNA.

Although amplification success decreased after 2 months, equid fecal piles persisted in the environment for longer than 6 months in our study site. Visual estimation of fecal age without any guidelines may lead to overestimation of time since deposition, which can lead to under sampling (Stenglein et al., 2010). In general, fecal pellets persist longer in dry environments (Harestad & Bunnell, 1987) and when sheltered (Lehmkuhl, Hansen, & Sloan, 1994), with exposure to rainfall hastening decay rates (Brinkman et al., 2009; Lehmkuhl et al., 1994). Perhaps because most studies used only fresh samples, few report how they qualify estimates of fecal age in the field. A noninvasive study of Sonoran pronghorn rated age on a three‐point scale (Woodruff et al., 2016), and a comprehensive study of elephant feces in a tropical forest environment described how it decayed under six categories (Barnes & Jensen, 1987). Deterioration of horse fecal samples in our study in Colorado, USA, seemed to occur in three stages over 6 months, based on coloration and texture: 1. Boli are green/brown and may be soft if very fresh; 2. Boli are mostly brown but begin to turn white; 3. Boli become white and begin to soften and deteriorate. From our results, equid feces that are mostly brown are likely to amplify sufficiently to yield genetic data.

The results from our study, that paper bags were more successful than ethanol vials for sample collection, that DNA from horse fecal samples amplifies well even 2 months after deposition, and that the best time to collect feces is during summer, suggest that there may be something about equid feces that predisposes it to be suitable for noninvasive sampling. As hind‐gut fermenters, equids spend about half their time feeding (King, Asa, Pluháček, Houpt, & Ransom, 2016; Schoenecker et al., 2016), and thus have an almost constant stream of food moving through their digestive system. This results in heavily mucosal feces providing an abundance of epithelial cells surrounding and/or throughout the fecal sample, such that even after some environmental degradation sufficient DNA remains for successful amplification. The size of a bolus of equid feces (normally at least 3 cm in diameter) and the fact that they are found in a clumped pile may act to shelter fecal DNA from degradation by UV light and precipitation. Furthermore, most equids are found in arid and semi‐arid ecosystems, which could promote faster drying rates and persistence of fecal boli. Although found in different parts of the world, equid species share a similar feeding ecology and use comparable habitat (Schoenecker et al., 2016). Thus, our analyses of feral horse fecal DNA are likely to be applicable across equid species for both management and conservation.

## CONFLICT OF INTEREST

None declared.

## AUTHOR CONTRIBUTIONS

Sarah King, Kate Schoenecker, and Sara Oyler‐McCance conceived the ideas and designed the methodology; Sarah King collected the data; Jenny Fike and Sara Oyler‐McCance conducted the genetic analyses; Sarah King and Sara Oyler‐McCance analyzed the data; Sarah King, Kate Schoenecker, Jenny Fike, and Sara Oyler‐McCance contributed to the drafting and revision of the manuscript and gave final approval for publication.
